# Analysis and implications of validation results for a clinical data-based early warning model for geriatric sepsis

**DOI:** 10.3389/fpubh.2026.1833804

**Published:** 2026-07-15

**Authors:** Xuejie Ma, Xinyi Li, Xiaoqiang He, Xin Zou, Xiaowei Ma

**Affiliations:** 1 Intensive Care Unit, Cardiocerebral Vascular Disease Hospital, General Hospital of Ningxia Medical University, Yinchuan, Ningxia, China; 2 First Clinical College of Ningxia Medical University, Yinchuan, Ningxia, China

**Keywords:** artificial intelligence (AI), older adults, machine learning (ML), model, sepsis, validation

## Abstract

**Objective:**

Sepsis poses a serious threat to the health and lives of older adults and is difficult to identify accurately in its early stages. To address this issue, our team previously developed an early warning model for sepsis in older adults based on machine learning algorithms and conducted preliminary validation. To further validate the model’s performance for clinical use, we collected additional clinical data. In the process, we gained some insights.

**Methods:**

We collected clinical data from patients aged 60 years or older admitted to the Emergency Department and Intensive Care Unit of the General Hospital of Ningxia Medical University. We conducted a secondary evaluation of the predictive and generalization capabilities of the geriatric sepsis sepsis early warning model using multiple metrics (including AUC, accuracy, sensitivity, specificity, precision, and F1 score), and analyzed the validation results.

**Results:**

Eight hundred fourteen patients were included in the study, comprising 106 positive patients (sepsis patients) and 708 negative patients (non-sepsis patients). After validation, the model’s accuracy was 0.801, sensitivity was 0.764, and AUROC was 0.865.

**Conclusion:**

In the study, although the AUROC of the further validation showed a slight decrease, the early warning model still performed well. The warning model is indeed helpful in identifying geriatric patients patients with sepsis earlier. The performance deviation observed during the validation phase may stem from multiple factors. Overall, machine learning models remain in a stage requiring continuous refinement and development. They are better suited as auxiliary tools in the medical field, necessitating long-term validation and adjustment rather than operating entirely independently.

## 1 Introduction

Population aging is the common challenge faced by both developing countries and developed countries (1, 2). Moreover, this issue is becoming increasingly prominent as societies progress (3). At present, China has a large number of older population in the world. By the end of 2024, in China, the population aged 60 and above exceeded 300 million for the first time, accounting for 22.0% of the total population (4). It is extremely necessary to pay more attention to the health issues of this group whose proportion is increasing at an ever-faster rate (5, 6). The trouble is that older population often suffer from multiple underlying diseases and have a weakened immune system (7). When they develop illnesses, symptoms frequently manifest inconspicuously or appear delayed relative to the onset of disease (8). Consequently, geriatric patients often experience delayed diagnosis due to the difficulty in recognizing their conditions. This leads to prolonged illness, resulting in poorer prognosis, severely threatening their quality of life, shortening their survival period, and simultaneously imposing heavy medical and financial burdens on both patients and their families. Sepsis, as one of the most common critical diseases in the intensive care unit (ICU) and emergency department, which is also a life-threatening condition for the older population (9), exhibits a marked increase in both incidence and mortality rates with advancing age (10, 11). For this reason, to address the critical clinical challenge of early identification difficulties, high mortality rates, and poor prognosis in geriatric sepsis patients, our research team previously developed an early warning model tailored to this specific population using clinical data from our hospital’s patients using machine learning algorithms (12).

Currently, the integration of artificial intelligence and medicine through machine learning methods to build diagnostic, predictive, and prognostic models has attracted widespread attention and research (13, 14). Numerous studies have shown that machine learning models that rely solely on a single round of internal validation have certain limitations (15–17). To optimize the accuracy and effectiveness of the model during application, it is prudent to conduct repeated validation of the model prior to deployment and implementation. Therefore, although our model performed well in the initial validation, in the subsequent research, we continuously optimized and further verified it, in an effort to make the model perform better in clinical settings. Based on the further verification results during this process, we have summarized the model’s performance once again and analyzed the findings. Through this study, we have summarized and analyzed the challenges encountered during the model development and validation process.

## 2 Materials and methods

In this study, patient data was collected by our research team using the same inclusion and exclusion criteria as during the model development phase so that the early warning model for sepsis in the older adults could be further validated. Patients were admitted to the emergency department and intensive care unit (ICU) from General Hospital of Ningxia Medical University. All of the patients were aged ≥60 years. None of these patients had received a sepsis diagnosis at the time of admission. The objective of this study is to predict the occurrence of sepsis in patients following a 24-h period of hospitalization.

During the model development process, we removed duplicate data, excluded features with a high proportion of missing values, encoded categorical variables, and filled in missing values using interpolation. Based on the dataset, categorical variables were encoded using label encoding, and ordinal variables were encoded using ordinal encoding. During the model validation phase, we did not perform data tuning on the validation dataset in order to better reflect the actual data conditions of patients in the real world. Model validation was conducted using Python 3.8.3 and scikit-learn version 1.3.2. We used grid search to tune the model’s hyperparameters: ‘n_estimators’: 300, ‘learning_rate’: 0.01, ‘max_depth’: 6. The hyperparameters used for model validation were consistent with those used during model training. For the XGBOOST machine learning method employed in the model, the number of trees was set to 300, and the maximum depth was set to 6.

In this study, we evaluated the predictive and generalization capabilities of the geriatric sepsis early warning model using various metrics, including the area under the receiver operating characteristic curve (AUROC), accuracy, sensitivity, specificity, precision, and F1 score.

## 3 Results

**Figure 1:**
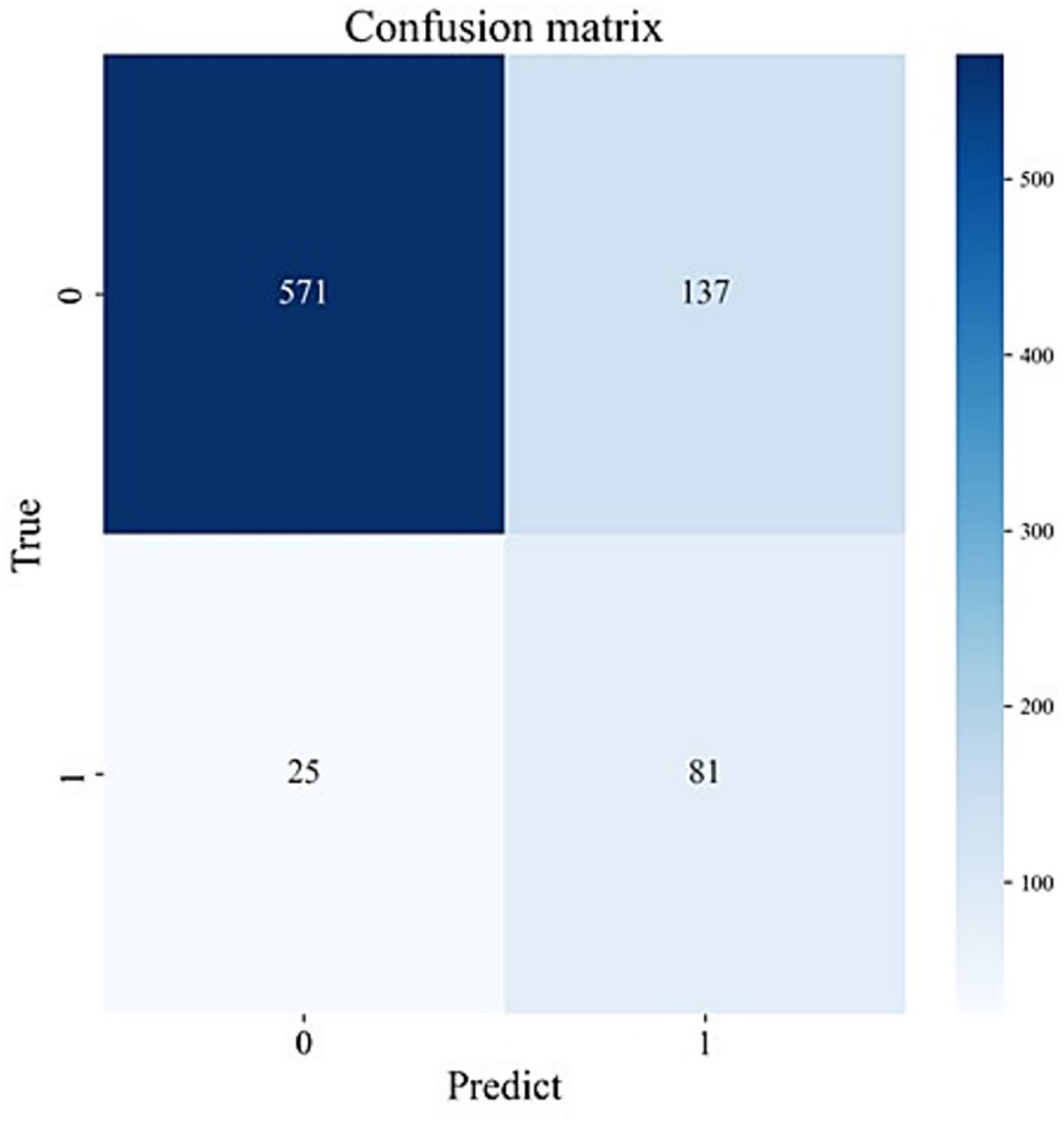
Confusion matrix of XGBoost model. The confusion matrix is a visualization tool used to evaluate the performance of machine learning classification models, particularly suited for binary classification problems. By plotting the confusion matrix, the predictive results of the early warning model for geriatric sepsis on new data are demonstrated.

**Figure 2:**
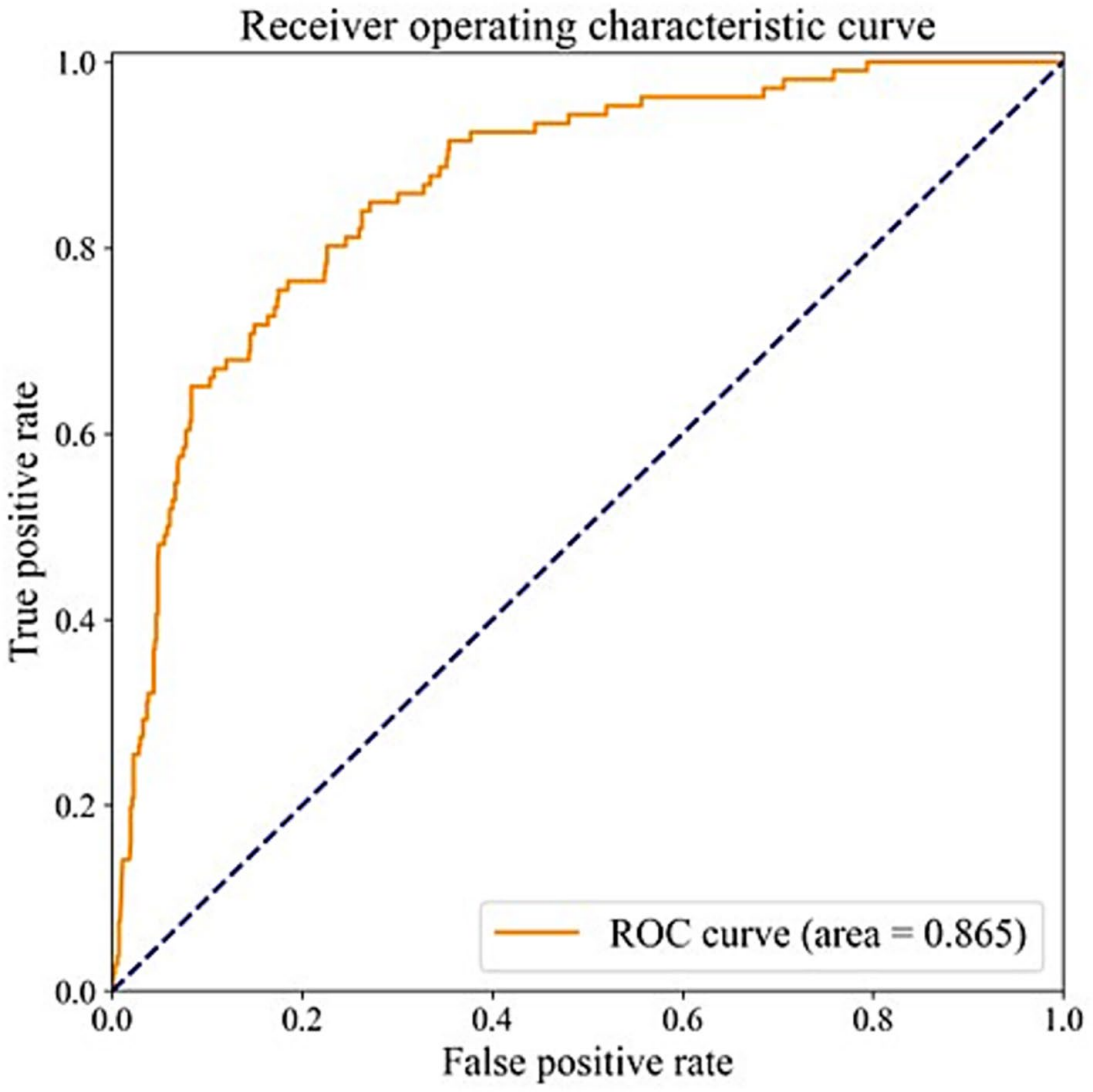
ROC curve of XGBoost model. The ROC curve generated by the early warning model for geriatric sepsis through this data validation has a false positive rate on the *x*-axis and a true positive rate on the *y*-axis.

Eight hundred fourteen patients from the emergency department and ICU in General Hospital of Ningxia Medical University were included in the study, comprising 106 positive patients (sepsis patients) and 708 negative patients (non-sepsis patients). The established early warning model for geriatric sepsis, which was built using XGBoost, was validated using these collected patient data. The predictive performance of the model was subsequently visualized through the utilization of a binary classification confusion matrix (see Figure 1). This method was employed to evaluate the performance of the machine learning model and to obtain classification metrics, such as sensitivity (see Table 1). Furthermore, we constructed a ROC curve (see Figure 2) and calculated the AUC to evaluate the model’s discriminatory capability. After validation, the model’s accuracy was 0.801, sensitivity was 0.764, and AUROC was 0.865.

**Table 1:** Evaluation metrics of XGBoost model.

Model	Accuracy	Sensitivity	Specificity	Precision	F1 score	AUROC
XGBoost	0.801 (0.773, 0.828)	0.764 (0.677, 0.847)	0.806 (0.777, 0.836)	0.372 (0.311, 0.436)	0.5 (0.432, 0.566)	0.865 (0.826, 0.896)

The relevant performance metrics are calculated for this model validation, including accuracy, sensitivity, specificity, precision, and F1 score. The model demonstrates well performance, attaining an accuracy of 0.801 and a sensitivity of 0.764. Furthermore, the AUROC is utilized to assess the model’s overall performance which was 0.865.

## 4 Discussion

In recent years, the issue of population aging has become increasingly prominent, with a growing proportion of older population (1). There is an urgent need to address health concerns among the older adults. Due to immune aging, the older adults are more susceptible to various bacterial infections (18). These infections often present with insidious onset, making early identification and diagnosis extremely challenging. Sepsis, a systemic inflammatory response syndrome triggered by infection, can lead to multiple organ damage, circulatory failure, and ultimately life-threatening complications (19). It ranks among the most common critical illnesses in intensive care units, characterized by rapid progression and high mortality rates, posing a serious threat to the lives and health of older adults (20, 21). At present, the pathogenesis and treatment methods of sepsis have been extensively studied (22). Regarding sepsis diagnosis, although multiple scoring systems assist in identifying sepsis but each has its certain shortcomings (23, 24). It is essential to comprehensively and dynamically assess patient conditions based on relevant indicators to achieve early recognition and potentially improve prognosis (25). This is particularly true for geriatric patients with sepsis, whose incidence and mortality rates increase with age.

AI is a field of computer science that aims to replicate human thought processes and decision-making capabilities through the development and implementation of sophisticated algorithms within computer systems (26). In recent years, there has been a significant increase in research activity concerning deep learning-based big data models that leverage AI technology in a variety of disciplines, including meteorology, biology, pharmaceuticals, and healthcare (27–30). Extensive research indicates that there are significant benefits to be gained from the integration of AI with the healthcare sector, particularly in the comprehensive analysis and evaluation of large-scale data sets. The development of sophisticated data models for medical applications, including but not limited to diagnosis (31), treatment (32) and prognosis (33), utilizing machine learning methodologies can effectively enhance medical practice standards. This development holds significant implications for the advancement of personalized medicine.

Given the characteristics of computer models and the advantages of contemporary big data models in healthcare, our goal is to optimize decision-making processes for geriatric sepsis by leveraging computer technology. By enhancing the ability to identify patients with geriatric sepsis, we aim to achieve early detection, diagnosis, and treatment to improve outcomes and enhance quality of life. The objective of our study was to develop a validated and highly accurate early machine warning model for geriatric patients with sepsis. Our research team constructed and validated the model using medical data from a large, comprehensive Grade A tertiary hospital in the Ningxia region. In preliminary research, we completed the model’s construction and initial validation. After comparing multiple machine learning approaches, this model was ultimately constructed based on XGBoost (12). After completing the initial modeling, we aim to make the model more suitable for clinical use through continuous optimization, adjustments, and further validation. As part of this ongoing process, we have incorporated additional real-world patient data to further validate the model as it is continuously optimized, evaluate its performance, and lay the groundwork for its deployment and application.

However, compared to the model-building phase, predictive ability has declined to some extent—a finding consistent with validation results from other machine learning models (34–37). This may disappoint many who have high expectations for AI models. Nevertheless, this phenomenon precisely highlights some of the problems involved in building machine learning models based on real-world clinical data. The decline in the model’s generalization ability, resulting from various factors, poses significant challenges for the model’s practical application. Current research always tends to emphasize the advantages of machine learning, sometimes may overlooking its limitations. When building models, the prevalence of the disease in the patient recruitment region may result in a significant disparity in the volume of data between the patient group and the non-patient group. This leads to class imbalance, which in turn affects the results (36). As demonstrated by our model, the number of sepsis patients is far lower than that of non-sepsis patients. Although the data composition used during model development accurately reflected the epidemiological situation in the region at that time, incidence rates vary depending on the region, the patient population at the hospital, and the time of onset (38–40). Therefore, the dataset used in the modeling process may no longer fully align with the incidence patterns of patients in the dataset during revalidation, leading to a certain degree of performance decline. Second, there are unavoidable issues with missing data during the data collection phase of the modeling process. Although efforts are made to minimize the proportion of missing data, it cannot be completely avoided (41). In addition, to ensure successful model development, methods such as median interpolation and multiple interpolation are commonly used to impute missing values (42, 43). Due to individual differences among patients, the interpolated data may deviate from the actual situation, thereby affecting the model results.

However, this does not negate the broad application prospects of machine learning models. Machine learning models do indeed offer numerous advantages. Machine learning models can process complex patient data more quickly than humans can, identifying key characteristics and alleviating the burden on healthcare workers (44). When applied to healthcare systems or extended to everyday tools such as mobile apps accessible to patients, these models can provide earlier and more timely health monitoring alerts. This will be very helpful in alleviating the burden of medical expenses.

Therefore, we should treat machine learning models as supplementary medical tools, rather than relying on them entirely. When using these models, we should exercise oversight and make judgments based on individual circumstances rather than placing absolute trust in them (45). Furthermore, we should strive to minimize outcome bias through various means. For instance, the model database is constantly updated, and the model is repeatedly adjusted based on the latest patient data. The performance of the model is dynamically monitored among the entire life of it. During model development, prioritize enhancing data quality over sheer volume to ensure that the data reflects real-world conditions as closely as possible.

## 5 Limitations of this model and areas for improvement

This research has some limitations. This model has currently undergone preliminary validation within a single healthcare system only, and has not yet been subjected to true external validation. Its applicability in external hospitals, different regions, and healthcare systems with significant differences in clinical practices has not yet been evaluated. This is the primary limitation of this study at this stage and represents an area requiring urgent breakthroughs in future research. Due to the epidemiology of sepsis in this region, the collected data sample suffers from class imbalance, with more negative cases than positive cases, resulting in low precision and a risk of false positives. Although sensitivity is prioritized over precision—as missed sepsis diagnoses can lead to irreversible organ damage or even death, particularly among frail older populations—low precision may indeed result in false positives, increasing clinical workload and causing potential disruptions, with a risk of alarm fatigue.

Our research is still ongoing. In the final clinical system, we will present the probability of disease occurrence rather than a simple alert, serving primarily as a tool to assist in early decision-making, with the final decision remaining the responsibility of the clinician’s comprehensive judgment. Future studies will focus on multicenter, prospective external validation. When applying and disseminating this model, it is necessary to first assess any biases in the data distribution. In the future, we plan to use local data to continuously and over the long term calibrate, update, or fine-tune the model. If necessary, we will establish mechanisms to continuously monitor the model’s performance to the greatest extent possible. Upon completion of this model validation, we plan to design a prospective multicenter study to systematically collect comprehensive data on SOFA, SIRS, and NEWS2 scores from the same patient cohort. We will then conduct a comprehensive comparison of this model against these scores within the same time window, evaluating discriminatory power, calibration, net classification improvement index, and decision curve analysis. We will continuously optimize the model performance. Our research group is working toward these goals, and we hope to achieving them as possible as we can.

## 6 Conclusion

In this study, we present the results of further validation conducted during the model optimization process; although the AUROC decreased, the early warning model under adjustment continued to perform well. Early warning models do indeed help identify geriatric patients with sepsis at an earlier stage. The performance deviation observed during the validation phase may stem from multiple factors. Overall, machine learning models remain in a stage requiring continuous refinement and development. They are better suited as auxiliary tools in the medical field, necessitating long-term validation and adjustment rather than operating entirely independently.

## Data Availability

The raw data supporting the conclusions of this article will be made available by the authors, without undue reservation.
